# The Discovery of the Venetian Blind Effect: A Translation of Münster (1941)

**DOI:** 10.1177/2041669517715475

**Published:** 2017-07-18

**Authors:** Edward T. Larkin, Wm Wren Stine

**Affiliations:** Department of Languages, Literatures, and Cultures, University of New Hampshire, Durham, NH, USA; Department of Psychology, University of New Hampshire, Durham, NH, USA

**Keywords:** Venetian blind effect, irradiation, stereopsis, luminance disparity, aniseikonia

## Abstract

Münster, the first to discover the effects of a luminance disparity on perceived depth, described two: (1) The apparent displacement in depth of one of a pair of objects relative to the other when viewed with a luminance disparity, and (2) The apparent overall displacement of objects viewed with a luminance disparity away from the observer. The first, which is the Venetian blind effect, was ascribed to irradiation. Current evidence suggests that irradiation fails to account for the effect, implying that neural mechanisms are involved. The second was thought to be related to the perceived distance of a monocularly viewed stimulus embedded in a dichoptically viewed stimulus. However, the measured effect was probably due to aniseikonia. Münster offered a compelling and seemingly complete account of the Venetian blind effect using irradiation theory. Münster’s irradiation theory effectively inhibited further research by relegating the perceived depth displacement to largely non-neural mechanisms. It is now becoming clear that Münster’s measurement of the Venetian blind effect represents the discovery of one of several mechanisms supporting stereopsis, though he and many others failed to recognize that discovery at the time.

## Introduction to Münster (1941)

Münster (1941) is the first paper describing, and explaining, what has become known as the Venetian blind effect, a term first coined by Cibis and Haber (1951). By virtue of the lateral displacement of one’s two eyes, each eye has a slightly different view of the environment, creating two-dimensional images on the two retinas that typically differ geometrically from one another. The visual system responds to such retinal disparities with the perception of depth, a process referred to as stereopsis. Imagine a case where two stimuli share a common fronto-parallel plane and so are a common distance from the viewer, such as two white rectangles drawn on a black background (Figure 1; as described by Münster, 1941). One will typically perceive these rectangles to occupy that plane based on their retinal disparities. If the rectangles are viewed with a neutral density filter in front of just one eye, thereby creating an interocular intensity disparity, the rectangles’ inner edges will appear to be in different depth planes. If the two rectangles are vertically oriented and their images fall on the central retina of each eye, they appear to be rotated about their individual axes, like two slats of a partially opened, vertically oriented Venetian blind. The Venetian blind effect is the apparent change in depth created by darkening the image on one retina relative to the other.

**Figure 1.**
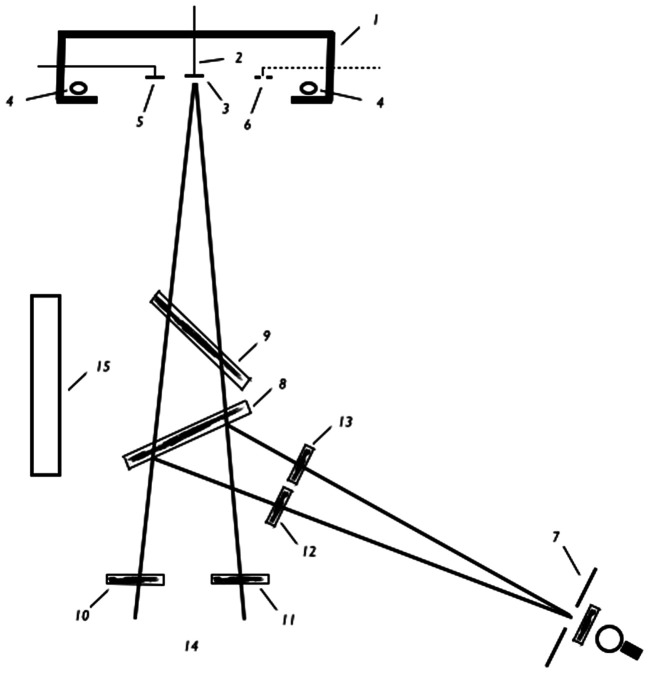
Schematic outline of the Horopter apparatus.

In that paper, Münster offers a compelling and seemingly complete account of the effect. As a result, we argue, further work on the effect was stifled; there was no more to be learned about stereopsis by studying the effect. Our accompanying translation of Münster (1941) reveals a paper written in a conversational style that was more common during the pre-war era, with little mathematical modeling. Contemporary empirical work on the Venetian blind effect, supported by modeling, demonstrates that Münster’s original account is essentially wrong, though his empirical results have largely been replicated, and certainly extended. After reviewing the Venetian blind effect with a brief introduction to the supporting empirical context, we describe the historical context leading to Münster’s discovery, and then build the argument that Münster’s explanation inhibited further study of the effect, thereby limiting the impact of his work.

Cibis and Haber’s (1951) use of the term Venetian blind effect, of course, results from the rotated appearance of rectangles whose images fall largely on the fovea. A Venetian-blind appearance also occurs when viewing vertical gratings dichoptically with a relatively large spatial frequency disparity (first described by Blakemore, 1970), which has also been called a Venetian-blind effect (Howard & Rogers, 1995, p. 259). Following Cibis and Haber (1951), we use the term to describe the effects of a luminance disparity on perceived depth.

## Empirical Context

### Münster’s (1941) Results

Münster (1941) developed a cancellation technique in order to measure the perceived shift in depth of stimulus edges when a pair of stimuli, similar to Figure 1, is viewed binocularly using a filter over one eye. His *horopter* apparatus allowed the subject to reposition one of the stimuli so that its edge matched in depth that of the other stimulus by moving one stimulus either toward the observer or further from the observer, thus cancelling the effect. Subjects used geometric disparities to cancel the perceived effects of luminance disparities.

Münster described his results using six “rules” encompassing two phenomena. Rule one, describing Phenomenon 1, stated that binocularly viewed edges appear to shift in depth with a luminance disparity, as though the edge’s image in the darkened eye shifted toward the light side of the edge (e.g., Münster, 1941, Table 2). Phenomenon 1 is the Venetian blind effect. The magnitude of Phenomenon 1 was quantified as the difference in the adjustment of the movable stimulus when one eye’s view was filtered relative to that when the other eye’s view was filtered.

**Table 2.** Typical Examples of the Dependence of Phenomenon 1 on Filter Absorptance and Type of Object.

Condition	a		b		c		d		e		f	
Object 1	White 60′		White 60′		Black 60′		Black 15′		Black 15′		Gray 15′	
	13 asb		13 asb								5 asb	
Object 2	White 60′		White 60′		Black 60′		White 60′		White 60′		Gray 60′	
	13 asb		13 asb				26 asb		26 asb		5 asb	
Background	Black		Black		White		Gray		Gray		Gray	
					28 asb		15 asb		7 asb		7 asb	
Filtered area	Total field		Object 2		Total field		Total field		Object 2		Total field	
Eye	l	r	l	r	l	r	l	r	l	r	l	r
65%	−32″	+36″	−11″	+17″	+29″	−17″	−8″	+7″	−17″	+6″	+0.5″	−4″
79.3	−52	+40	−42	+13			−24	+15	−27	+9	−9	−17
87.1	−72	+55	−38	−6	+26	−27	−35	+18	−45	+28	−18	−15
92.8	−53	+60	−28	−9			−47	+40	−55	+40	−19	−26
95.7	−97	+62	−66	−22	+29	−42	−52	+41	−68	+45	−31	−42

Phenomenon 2 was described by rule two. Objects appear slightly more distant from the observer when viewed with a luminance disparity, giving a deviation from zero in the geometric disparities required to cancel the apparent edge shifts in depth when they are averaged across the left and right eyes (e.g., Münster, 1941, Table 2 Conditions a and b, Table 5 Series b).

**Table 5.** Examples of the Dependence of Phenomenon 2 on Filtering.

Test Series	a		b		c		d	
Object 1	Black 15′		White 60′		Gray 15′		Black 15′	
	14 asb		5 asb					
Object 2	White 5′		White 60′		Gray 15′		White 5′	
	11 asb		14 asb		5 asb		11 asb	
Background	White		Black		White		White	
	7 asb		7 asb		7 asb			
Filtered Region	Object 2		Object 2		Total Field		Total Field	
Filter Absorptance	l	r	l	r	l	r	l	r
65%	−15″	−18″	−11″	+17″	−10″	−12″	−6″	−6″
79.8	−32	−8	−42	+13	−19	−30	−22	−2
92.8	−43	−32	−28	−9	−39	−40	−38	−18
95.7	−38	−37	−66	−21	−49	−44	−64	−36

#### Phenomenon 1: The Venetian blind effect

Three results, or rules, were asserted for Phenomenon 1 (Münster, 1941, Rules 3–5):

- Increasing luminance disparity increases the effect (Münster, 1941, Table 2, Rule 3), which was replicated by Cibis and Haber (1951, Figures 6 and 7), Hetley and Stine (2011, Figure 5), and, indirectly, Filley, Khutoryansky, Dobias, and Stine (2011, Figure 4).
- As the width of the filtered objects increase from 5 to 60 minutes of arc, the effect at first increases but then remains constant (Münster, 1941, Table 3, Rule 4). Filley et al. (2011, Figure 11f) found a slightly *decreased* perceived rotation with bar widths from 11.7 to 27.4 minutes, while Khutoryansky (2000, Figure 5, *Investigation of influence of blurry edges on Venetian blind effect*, Unpublished Master’s Thesis, University of New Hampshire) found no effect of bar width on threshold luminance disparity to see a rotation (7.70 to 33.0 minutes of arc). Both studies used periodic stimuli in Maxwellian view with luminance levels roughly five times those of Münster’s.
- The effect increases with the contrast between the object and the background (Münster, 1941, Tables 2 and 4, Rule 5), which no one has tried to replicate.

**Table 3.** Examples of the Dependence of Phenomenon 1 on the Horizontal Retinal Angle of the Compared Objects.

Test Series		a		b		c		d	
Object 1		White		Black		Black		White 1^0^	
		15 asb						15 asb	
Object 2		White		Black		White		White 1^0^	
		15 asb				26 asb		15 asb	
Background		Black		White		Gray		Black	
				28 asb		15 asb			
Total Field									
95.7% Absorbance									
Object size									
1	2	l	r	l	r	l	r	l	r
5′	5′					−37″	+3″		
5′	15′	−69″	+20″	+7″	−12″	−34	+32		
5′	30′					−67	+14		
5′	60′					−52	+41		
15′	15′							−69″	+20″
30′	30′			+14	−27				
60′	15′	−75	+37						
60′	30′	−94	+56						
60′	60′	−78	±64	±23	−32			−99	±62

**Table 4.** Examples of the Dependence of Phenomenon 1 on Contrast.

Test Series	a		b		c		d		e	
Object 1	Black 30′		Black 30′		Black 30′		Gray 60′		Black 15′	
Object 2	Black 30′		Black 30′		Black 30′		White 60′		White 60′	
Background	White		White		White		Gray		Gray	
Filter	95.7%		87.1%		65%		87.1%		95.7%	
Contrast	l	r	l	r	l	r	l	r	l	r
Object:Background										
0:20	+2″	−9″	+1″	−21″	+3″	−19″				
0:360	+14	−40	+12	−35	+9	−16				
26:20							−14″	+29″		
50:20							−49	+27	−11″	+27″
120:20							−82	−1.5	−42	+32
720:20							−63	±26	−52	±41

#### Phenomenon 2

In the situation where two white stimuli are presented before a black background, the average disparity across multiple adjustments with a filter over the left eye and then the right eye was found to be non-zero (e.g., Münster, 1941, Table 2 Conditions a and b, Table 5 Series b), with larger crossed disparities required to null perceived depth than uncrossed disparities.

Interestingly, Cibis and Haber (1951, Figure 7) replicated Phenomenon 2 when data were averaged across 10 subjects, with one subject showing a very large effect (Cibis & Haber, 1951, MH in Figure 7). However, while roughly half of the subjects demonstrated a smaller shift when the right eye was filtered than when the left eye was filtered, the remaining subjects showed the opposite.

Phenomenon 2 yielded just one result for Münster (1941, Table 5, Rule 6): The magnitude of the effect increases as the density of the filter increases.

### Münster’s (1941) Theory

#### Phenomenon 1: The Venetian blind effect

Münster (1941) states that irradiation accounts for Phenomenon 1. When viewing, for example, a white rectangle on a black background, irradiation (von Helmholtz, 1911/1924, pp. 188–189; Galileo also recognized the phenomenon, Brown, 1985) describes the effects of the imperfect optics of the eye, coupled with the compressive non-linear processing of the visual response to light intensity, on the perceived location of the rectangle’s edge that separates the light and dark regions. Briefly, the eye’s optics are imperfect. So the image of a sharp edge on the retina will be blurred. The visual response to light intensity can be characterized using a compressive non-linearity. Hence, the visual response to a blurred edge will be distorted compressively. If the edge separates a light from a dark region and the perceived location of that edge is defined by where the visual response equals a particular value, the compressive non-linearity will shift the location of that value, and so the perceived location of the edge, toward the dark region. A white rectangle on a black background will thus appear larger than a black rectangle of exactly the same size on a white background. As well, the bright region will appear to grow at the expense of the dark as the luminance of the bright region increases (about 0.4 minutes of arc when viewing a stimulus with a 113 cd/m^2^ bright region bordering a region of less than 1 cd/m^2^; Westheimer, 2007). In the filtered eye, the apparent movement will be smaller than in the unfiltered eye, creating a perceived geometric disparity. Münster’s stimuli would be expected to generate considerably less movement than the 1.0 minutes shift he measured. Further, Münster (1941, Table 2, conditions a vs. b) found that if the whole field is filtered a larger effect is seen than if only the region immediately surrounding the object is filtered, which is not predicted by irradiation.

Current evidence suggests that irradiation provides a poor account of the Venetian blind effect: (a) The results when manipulating the luminance and contrast disparities of stimuli do not match that predicted by irradiation (Filley et al., 2011, Experiment 1; Münster, 1941, Table 2, conditions a vs. b), (b) the results expected from blurring the edge between light and dark regions are not obtained (Filley et al., 2011, Experiments 2 and 3), and (c) the temporal dynamics of the Venetian blind effect do not match those of a geometric disparity, as predicted by irradiation (Dobias & Stine, 2012).

#### Phenomenon 2

Münster (1941) related Phenomenon 2, the displacement of a target away from the observer when viewed with a luminance disparity, to viewing an object monocularly that is embedded in a binocular field of view. He asserts that, if one views a stimulus dichoptically with zero disparity, and then inserts a second stimulus into only one eye’s view, the second stimulus will appear to be slightly further away from the observer than the first. Münster (1941) reasoned that this configuration is the limit of viewing a stimulus dichoptically with neutral density filters of increasing opacity interposed between one eye’s view and that eye’s stimulus; for a very high-density filter, one eye’s view of the dichoptically presented stimulus vanishes, resulting in a monocularly viewed object placed in a binocular field of view. A special case of this stimulus configuration is that used to demonstrate da Vinci stereopsis (Nakayama & Shimojo, 1990; Tsirlin, Wilcox, & Allison, 2012).

Considering that a stimulus will appear darker when viewed binocularly with a neutral density filter over one eye than when viewed without the filter (e.g., De Silva & Bartley, 1930; Fry & Bartley, 1933; Hetley & Stine, 2011) and that a darker stimulus typically appears to be further away than a brighter stimulus (e.g., von Helmholtz, 1911/1924, p. 187, Figure 32; Westheimer, 2007, Figure 1), Münster’s reasoning sounds plausible. However, both the adjustable stimulus, on the left, and the standard stimulus, on the right, will look further away when viewed binocularly with the filter over just one eye since both eyes view the entire stimulus array. So, the average adjustment to cancel the effect should be zero when using Münster’s horopter apparatus.

Given that Münster’s (1941) Phenomenon 2 is measured as a shift in the mean geometric disparity required to cancel perceived depth across conditions that should be balanced, our view is that this effect probably reflects aniseikonia, or “unequal vision” (an interocular difference in the perceived size of images; Cibis & Haber, 1951; Ogle, 1952) in the subject used in his study. Aniseikonia can be measured by asking the observer to cancel perceived rotation or depth with no luminance disparity, which Münster (1941) never performed. A non-zero geometric disparity setting would indicate aniseikonia. As mentioned previously, one of Cibis and Haber’s (1951, MH in Figure 7) subjects exhibited relatively strong aniseikonia (see also Hetley and Stine, 2011, Appendix).

### Summary

Münster’s (1941) Phenomenon 1, the Venetian blind effect, yielded results that were measured quantitatively and were generally replicated. His explanation for the effect, irradiation, qualitatively accounts for his basic results. However, some of Münster’s results and contemporary results, as noted, contradict the irradiation account of the Venetian blind effect.

Phenomenon 2, that viewing an object with an intensity disparity increases the apparent distance to that object, has been replicated in some subjects by other researchers. Other subjects show the opposite effect, suggesting that Phenomenon 1 may be due to aniseikonia. In the following, we will focus on just the Venetian blind effect, Phenomenon 1.

## Historical Context

At the time of his discovery, Münster was working for Carl Zeiss Jena as Director of Development. One other effect had been discovered at Zeiss Jena 19 years before Münster’s work. Carl P. Pulfrich (1858–1927) published an article (Pulfrich, 1922) in six parts that describes what is now known as the Pulfrich effect with an application of that effect to measuring the intensity of a light source psychophysically. As with the Venetian blind effect, to generate the Pulfrich effect one views a stimulus using both eyes but with a dark piece of glass over just one eye, again creating an interocular intensity disparity. The stimulus, however, is typically a pendulum that is swinging in a fronto-parallel plane. When viewed with an intensity disparity, the swinging pendulum appears to leave the fronto-parallel plane and swing in a roughly elliptical path in depth. If the right eye is viewing the pendulum through the filter, the pendulum will appear to deviate toward the viewer when passing left to right and away from the observer when passing from right to left. Current evidence suggests that the processing of the visual system slows as the intensity of the image being processed drops. Hence, the eye viewing the swinging pendulum through the filter processes that movement more slowly than the eye viewing the pendulum directly, resulting in a geometric interocular disparity between the perceived positions of the swinging pendulum at any given moment (Lit, 1949; Rogers & Anstis, 1972; Williams & Lit, 1983). The pendulum is thus perceived to leave the fronto-parallel plane (however, the apparent path does not form an actual ellipse given a constant interocular response latency with a constant intensity disparity; Ogle, 1962, p. 300).

Pulfrich, having earned his PhD in 1881 from the University of Bonn, joined the Carl Zeiss Jena optics company in the 1890 where he remained until his death by drowning in a canoe accident (see Christianson & Hofstetter, 1972, for a brief biography of Pulfrich). According to Christianson and Hofstetter (1972), Pulfrich’s papers focused on refractometry (measuring the refractive index of a substance, which is useful for assessing purity, etc.) from 1885 to 1899, stereoscopy (presenting two images, one to each eye, that differ geometrically, which was used to detect change in two pictures taken sequentially, for example) from 1899 to 1920, and photometry (measuring the intensity of a stimulus weighted by the human spectral sensitivity function) from 1920 to 1927, the period that included the discovery of the Pulfrich effect. Pulfrich (1922) reports that the change in perceived depth with movement during stereocomparitor measurements was first reported by Wolf in 1920. Zeiss gave the problem to an engineer, Franke, and a technical assistant, Fertsch, to explore since the change in depth could be construed as a problem with Zeiss-made comparators. Apparently, Fertsch initially discovered that an intensity disparity coupled with motion created the effect. Pulfrich’s (1922) original contribution was to adapt the effect to the measurement of the intensity of two stimuli that differed in wavelength composition (heterochromatic photometry; Lit, 1949). Clearly, by the early 1920s, the research staff at Zeiss was very interested in stereoscopy and the effects of interocular intensity differences on perceived depth.

Clemens Münster (1906–1998) was born in Cochem on the Mosel, Germany. He studied physics, mathematics, and chemistry in Münster and Munich from 1924 to 1928. After receiving his doctorate in optics, he spent 2 years at the Institute for Applied Optics, University of Jena, and then moved to the Physics Institute of the University of Bonn. In 1934, Münster began working for Carl Zeiss Jena, where he was Director of Development until 1945.

We know of no record indicating that Münster either met, or even knew of, Pulfrich. However, given the attention that the Pulfrich effect drew (e.g., Engelking & Poos, 1924; von Kries, 1923) and that both Pulfrich and Münster worked for Zeiss, it seems highly likely that Münster was familiar with Pulfrich’s work. Indeed, given that Münster was within a year of the PhD when Pulfrich died, they very well may have met.

## Impact

As mentioned, Münster (1941) offered an irradiation account of the Venetian blind effect. As a qualitative theory of the effect, irradiation appears complete. Further, since the theory posits a role for just the eye’s optics and the compressive non-linearity of the visual system’s response to intensity, it would appear that little could be gained in our knowledge of stereopsis by studying the effect. Indeed, when Cibis and Haber (1951) rediscovered the effect, in ignorance of Münster (1941), and developed their irradiation account, Ogle (1952) wrote that “Their experiment cannot therefore be used to imply either a special type of spatial perception or a new anomaly in the process of binocular vision” (p. 142). An irradiation model removes the Venetian blind effect from the list of possible effects involving stereopsis; and so is not worth pursuing if one wishes to study stereopsis.

Some papers have appeared over the intervening years that mention the Venetian blind effect, though few have considered stereopsis per se. Miles (1953, 1954), citing just Cibis and Haber (1951), explored applied topics briefly, such as the impact of the Venetian blind effect on pilots, and presented what he claimed to be a new phenomenon that he called anisodominance, which was defined as the effect of ocular dominance on perceived distance to each of a pair of stimuli presented on opposite sides of fixation. Neither of Miles’s two papers explored the Venetian blind effect empirically. Miles also coined the term irradiation stereoscopy to describe manipulating retinal disparities through irradiation effects, a term later used by Ogle (1962, pp. 302–303, who did not cite Miles; see also Howard & Rogers, 1995, p. 310; Howard & Rogers, 2012, p. 286).

Ogle (1962, pp. 302–303) appears to be the first to have cited both Münster (1941) and Cibis and Haber (1951), noting that their discoveries were independent and mentioning that the effect might prove useful in the study of the eye’s optical quality. The initial wave of quantitative image quality measurements on the retina were conducted in the late 1950s and early 1960s (e.g., Campbell & Gubisch, 1966; Flamant, 1955; Krauskopf, 1962; Röhler, 1962; Westheimer, 1963; Westheimer & Campbell, 1962), using techniques that differed from the indirect approach through the Venetian blind effect suggested by Ogle (1962, pp. 302–303). Interestingly, these researchers might have recognized that Münster’s Phenomenon 1, the Venetian blind effect, represented the discovery of an unnoticed mechanism of stereopsis had their results been applied to quantitatively model irradiation in the context of an intensity disparity (as was done by Filley et al., 2011).

Von Békésy (1970), citing Münster (1941), Cibis and Haber (1951), and Ogle (1962, pp. 302–303), was the first to integrate measurements of image quality in the eye with the irradiation theory of the Venetian blind effect. Demonstrating that stimulus configuration influences the degree of perceived rotation when a stimulus is viewed with an intensity disparity, von Békésy concluded that lateral inhibition decreased the effects of irradiation, and that perceived rotation was the result of a mixture of irradiation and lateral inhibition. Fiorentini and Maffei (1971) then used sine-wave gratings surrounded by a black aperture and viewed with a contrast disparity to demonstrate perceived rotation without geometric or intensity disparities. The spatial characteristics of sine-wave gratings with a contrast disparity should not be influenced by irradiation, though portions of the black aperture might be subject to irradiation with a contrast disparity. At any rate, Blake and Cormack (1979) failed to replicate Fiorentini and Maffei (1971). Walker (1976) measured the relative contributions of the Venetian blind effect and the Pulfrich effect to the perceived slant of rotating, textured disks with the resulting aftereffects. His results were interpreted in the context of irradiation and the effects of image intensity on visual processing speed. Kumar (1995) presented a set of five demonstrations showing depth effects with interocular luminance differences restricted to small regions of the stimuli where, it was claimed, irradiation should have negligible effects (see, also, Howard & Rogers, 1995, pp. 310–311). Neither Fiorentini and Maffei (1971) nor Kumar (1995) actually modeled irradiation effects for the stimuli that they used.

So, the first paper to suggest that the Venetian blind effect may reflect more than what has been called irradiation stereoscopy was von Békésy (1970), 29 years after the effect’s discovery and 19 years after its re-discovery. It was another 41 years before a series of papers appeared that clearly refuted the proposition that irradiation theory accounts for the Venetian blind effect (Dobias & Stine, 2012; Filley et al., 2011; Gillam & Wardle, 2013; Hetley & Stine, 2011; Lappin, 2014). The few papers that did mention Münster (1941) or Cibis and Haber (1951) before 2010, other than von Békésy (1970), assume irradiation as a complete account (Fiorentini and Maffei, 1971; Howard & Rogers, 1995, pp. 310–311; Kumar, 1995; Miles, 1953, 1954; Ogle, 1962, pp. 302–303; and Walker, 1976; see also Howard & Rogers, 2012, p. 286).

## Conclusion

Münster’s (1941) compelling and seemingly complete account of the effect using irradiation would seem to have had a devastating effect on continuing work in this area. That Cibis and Haber (1951) generated a very similar theory upon their rediscovery of the effect indicates just how compelling, and natural, such a theory seemed. Even after the optics of the eye had been mathematically characterized by the early 60s, only one paper, von Békésy (1970), approached irradiation and the Venetian blind effect quantitatively for nearly 50 years.

That multiple cortical channels are involved in stereopsis is well established (see Parker, 2007). That disparities across multiple dimensions support stereopsis (luminance: Münster, 1941; Cibis & Haber, 1951; contrast: Filley et al., 2011; Fiorentini and Maffei, 1971—but see Blake and Cormack, 1979; Hetley and Stine, 2011; motion supporting motion-in-depth: Nefs, O’Hare, & Harris, 2010; contrast summation: e.g., Baker, Meese, & Georgeson, 2007; Hetley & Stine, 2011; Meese, Georgeson, & Baker, 2006, which seems to survive in the face of amblyopia; Baker et al., 2007) is becoming increasingly clear. Münster’s scientific legacy, unrecognized for 70 years, may entail the initial discovery of one of several mechanisms supporting stereopsis.[Fig fig2-2041669517715475]

**Figure 1. fig2-2041669517715475:**
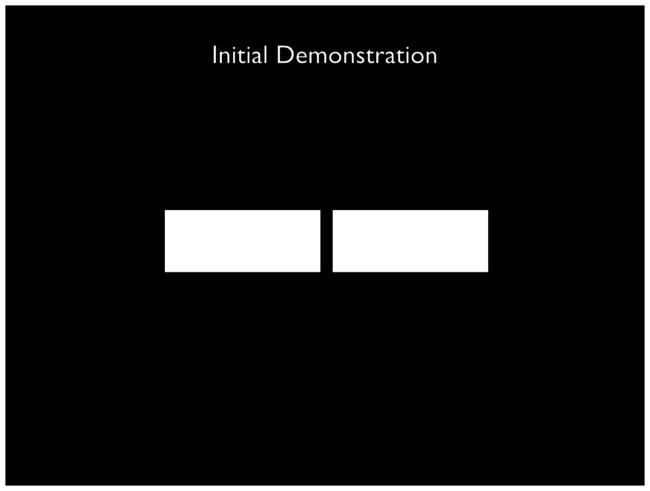
A rendition of the first stimulus described by Münster (1941). Viewed binocularly with a neutral density filter over the left eye, the rectangle on the left will appear recessed in depth relative to the one on the right. If the right eye peers through the filter, depth is reversed.

## The Translation: The Effect of Interocular Brightness Differences on Stereoscopic Perception

### Cl. Münster (deceased)

### Introduction

During a systematic study of the factors that cause a change in normal stereopsis, the question arose whether or not an interocular difference in brightness had an effect on stereoscopic depth localization. An interocular difference in brightness can impact the whole field of vision, which may include the perceived depth of the objects to be compared relative to their background, or the objects relative to one another and apart from the background, or the difference can impact only some of the objects located in the field of vision. An interocular difference in brightness can be artificially produced by interposing a filter between one eye and its object or by varying the luminance of the presented images. It can also result from a disparity in the sensitivity of the eyes.

By means of a simple experiment, it can be demonstrated that an interocular difference in brightness can bring about changes in spatial perception: If one cuts two rectangles from a sheet of black paper approximately 50 mm by 20 mm and then affixes the sheet to a piece of silk paper on a pane of glass such that the openings are similarly illuminated, and if one then places a neutral density filter with an absorbance of at least 50% in front of the left eye, the left edge of the inner pair of edges seems to recede. If the filter is placed in front of the right eye, the phenomenon is reversed.

Experiments on the effect of placing a filter over one eye have thus far been carried out only in connection with the Pulfrich (1922)^1^ effect and by Verhoeff (1933a, 1933b).

The Pulfrich effect occurs when an object in motion is observed binocularly as the luminance of the object viewed by one eye is reduced by a filter.The effect is based on the relationship between the duration of perception and the intensity of a stimulus.

The basic experiment by Verhoeff (1933a, 1933b) is as follows: Each eye is presented with a black line on a white background; the two lines slant slightly toward each other. The binocularly fused image seems then to run from the upper front to the lower back in the medial plane or vice versa. If the retinal illuminance of one eye is decreased, a distinct flattening of the spatial impression and a simultaneous outward movement from the medial plane of the fused pattern appears, and this occurs in such a way that the fused image approaches the appearance of the image viewed by the eye whose retinal illuminance was not decreased. This and other experiments conducted by Verhoeff are linked to the presence of retinal disparities. The flattening of the spatial impression corresponds exactly to the decrease of retinal disparity, bringing about the apparent tilt. The experiments show that the unfiltered eye dominates over the filtered eye.

The present essay reports on a study of the effect of an interocular difference in retinal illuminance on the stereoscopically perceived relative depth of resting objects with zero disparity, that is, on the placement of two objects at apparently equal distances from the observer.

### The Configuration of the Experiment

The experiments were conducted on the horopter^2^ apparatus reproduced in Figure 1 (schematic outline). (1) is a box with a white interior and a black exterior, from whose front wall a rectangular opening of about one square meter was cut. On the back wall there is a hole into which a guide stick (2) is inserted; a surface (3) is attached to the tip of the stick making the stick invisible to the human subject at (14). A chin support keeps the subject’s head in place. The subject can adjust the depth of the guide stick (2) and the object (3), by means of two strings that run on rollers (not pictured in the illustration). A ruler measures the change in position in millimeters; it is attached to the stick (2) and is projected onto a ground-glass plate. Illumination occurs by means of soffit lamps (4), hidden from the subject, and is practically without shadow. A fixed surface can be attached at (5) or (6). (3), (5), and (6) are cut out of a piece of tin and are painted flat black, white, or gray. An additional light-colored surface on a dark background can be attached at (7) and reflected into the subject’s eyes (14) through the half-silvered mirror beamsplitter (8), such that it can be seen in the vicinity of surface (3). Illumination of surface (7) occurs through a milk-glass pane by means of a lamp that is screened from the subject. The half-silvered mirror beamsplitter (9) can also be attached in order to superimpose the luminance of the uniform white surface (15) onto the field of vision, such that, for example, a black surface (3) appears gray. To filter the retina of one eye, a suitable neutral density filter is used; it can filter the whole field of vision of the left eye of the subject when placed at position (10) or that of only the right eye when placed at position (11). Only the image of the surface (7) is filtered for the left eye when the filter is placed at (12) or for the right eye when placed at (13).

By means of the sequence described, the following scenarios can be observed:

- Two black objects on a white background: surfaces (3) and (5) or (6) without the mirrors (8) and (9).
- Two gray objects on a white background: surfaces (3) and (5) or (6) and mirror (9).
- Two white objects on a black background: surface (3) is replaced by a large metal sheet painted flat black, from which a corresponding opening is cut; surface (7) and mirror (8).
- Two white objects on a gray background: like (III), but with mirror (9).
- One black and one gray object: surface (3) black, surface (5) or (6) gray.
- One black and one white object: surfaces (3) and (7).
- One white and one gray object: like (VI), but with mirror (9).

The relative position of the objects to one another can be easily changed, vertically or horizontally, by moving objects (5), (6), or (7).

Five natural density filters were used with the following absorbances: 65%, 79.3%, 87.1%, 92.8%, and 95.7%. Even the effects of this final filter could be measured under the experimental conditions.

The neutral density filters and mirrors that were interposed between the eye and the object were of outstanding optical quality and precluded any distortion. The overall configuration of the experiment satisfied the usual requirement that the impact of secondary cues on depth localization be limited.

The distance of the subject from the middle surface (3) was 3.75 m. At this distance and given an interpupillary distance of 68 mm, a shift of 1 mm in the movable surface (3) corresponds to a change in retinal disparity of one arc second.

### Procedure

In all seven of the scenarios noted above, the impact of the filtering of one eye on the perceived depth of various objects was studied. The variables were: the filter, the lateral position of the objects to one another, the luminance contrast between object and background, and the size of the objects. This resulted in several variations of the following task: The movable surface (3) was to be set in such a way that a difference in depth between it and the fixed surfaces (4), (5), or (7) was no longer perceptible. When the setting of surface 3 deviated from the position in which the objective disparity of both stimuli was zero, the newly determined disparity had the same value as the effect of the difference in retinal illuminance.

Each measurement consisted of 10 settings, which were then averaged. The relative error varied with the size of the setting, between 2 and 8 seconds of disparity, but was usually under 4 seconds.

All of the experiments were carried out with a subject who had normal vision, some experience in making measurements of this kind, and equal light sensitivity in both eyes. The more important experiments were repeated with several subjects and produced essentially the same results.

The measurements were taken in a darkened room. We did not wait for both eyes to completely adapt to the darkness since that would not have had any significant effect on the measurements of the kind described. The adaptation of the filtered eye, especially when the whole field of vision was filtered, was of course different from that of the unfiltered eye. Here too, complete adaptation was not obtained. This could be seen on occasion in some of the experiments in the course of a series of 10 settings: The initial results were somewhat greater than those taken later. But these deviations were only on the order of a few seconds of disparity and so could be neglected.

### Results

As the experiments show, the effect of a retinal illuminance disparity on stereopsis can be summarized in a few rules.

#### Rule 1

If the field of view for the left and right eyes differ in luminance, the edges of the objects viewed in the darker field of view—and the edges are crucial for relative depth localization—always appear to shift toward the lighter areas of the image. We call this effect Phenomenon 1. It is certainly reproducible and easy to recognize.

Table 1 presents an overview of Phenomenon 1 when two objects are in play. One can first of all see how the perceived relative position of the two objects changes when the left or the right eye is filtered. The edges are formed by (a) a light object on a dark background and (b) a dark object on a light background. The physical changes in the position of the two images of the objects in the filtered eye that are indicated by variable “s” would create the perceived relative change in the positions of each object indicated by variable “t,” creating the same relative change in depth as the filtering actually brought about. In accordance with Rule 1, the apparent relative change of depth, as a consequence of the filtering of one eye, is equivalent to moving the two images of the objects apart in the filtered eye if the objects presented are light on a dark background; on the other hand, if the objects presented are dark on a light background, their images appear to move toward each other. The sign of the apparent change of depth, effected by Phenomenon 1, therefore, is changed either if the filtering of the right and left eyes is interchanged or if the luminance ratios between the objects and the background is reversed. In accordance with the apparent change in relative depth for two objects lying next to each other, Phenomenon 1, by and large, disappears when the objects are placed one above the other.

**Table 1.** Overview of Phenomenon 1.

	Left eye filtered				Right eye filtered			
	Light object		Dark object		Light object		Dark object	
	Dark background		Light background		Dark background		Light background	
	T	s	t	s	t	s	t	s
Left Obj:	Back	Apart	Forward	Together	Forward	Together	Back	Apart^a^
Right Obj:	Forward	Together	Back	Apart	Back	Apart	Forward	Together

*Note*: Objects above one another: No effect. t = Observed apparent relative depth change of both objects; s = Corresponding relative lateral shift of both objects’ images viewed by the filtered eye.

a Translator’s footnote: The author used “zusammen,” meaning “together,” in the original article, and we have assumed that this is an oversight since the actual corresponding shift of the edges is “apart.”

#### Rule 2

There is a definite tendency to localize a single object differently—and generally at a farther distance—if one eye’s field of view is less well illuminated than the other. If both fields of view are equally illuminated, then an object placed at the same physical distance as that in the first scenario would appear to be closer.

This effect shall be designated Phenomenon 2. It does not always occur; it is rarely reproducible by virtue of its magnitude, and it is more difficult to recognize than Phenomenon 1, which it overlaps when the two objects are located adjacent to one another.

If, as per the first rule, one does not use a neutral density filter at positions (10) or (11) but rather places it at positions (12) or (13) in illustration 1 so that, instead of the whole field of vision being filtered, only object (7) is filtered for the left eye or the right eye, then Phenomenon 1 is again observed. But the mean of the settings created when one eye’s view is filtered—left or right—no longer coincides with that of the settings when the eyes are unfiltered but, in general, is shifted back in depth. That is normal when Phenomena 1 and 2 are brought together.

Phenomenon 2 is especially noticeable if one uses a very strong filter for an object; ultimately, the image of this object completely disappears for the filtered eye. In this case, a monocularly seen object (usually, but not always) appears to be shifted significantly backwards vis-à-vis a binocularly seen object.

Apart from the overlap of Phenomena 1 and 2, the results are further complicated by the fact that, in addition to artificially created interocular differences in luminance sensitivity, there are also natural differences. The principal experiments were carried out on a subject whose sensitivity to luminance was practically equal in the left and the right eye when both had adapted to the given luminance. But the consequences of adaptation to luminance differences cannot be ignored. Their impact on the measurements has already been noted. In addition, many observations led us to suppose that the luminance sensitivity of the retina of each eye is subject to spatial and temporal fluctuations. All of these influences are also evident in the measurements taken on other subjects. The results showed that the two rules listed above (and the contingency of Phenomena 1 and 2 on several factors) retained their qualitative validity for all subjects. But the measurable size of both phenomena and the degree of overlap showed greater fluctuation among the experimental subjects.

In spite of the noted complications, certain relationships in the experiment can be identified with sufficient certainty. The results of the measurements conveyed in the following tables are, however, understandably only typical examples taken from a considerably larger set of data.

In Tables 2–5, the “apparent relative changes of depth” between two objects (1) and (2) are given in arc seconds of disparity for the experimental Columns “a” to “f.” The numbers represent the means taken from 10 settings, and the functional signs refer to the object seen on the left by the observer: A positive sign indicates that this object appeared closer in depth than the right object, and a negative sign indicates that it appeared further in depth than the right object. “l” indicates that the left eye was filtered; “r” indicates that the right eye was filtered. The filtering is given in percent absorbance; the luminance is given in Apostilb, and the disparity of the object is given in arc seconds^3^ with respect to the distance from the observer. Phenomenon 1 is measured by the difference between the measurements that were taken when the left eye was filtered and when the right eye was filtered. Phenomenon 2 is measured by the deviation from zero of the mean of the measurements that were taken when the left eye was filtered and when the right eye was filtered.

#### Rule 3

Phenomenon 1 increases with increasing differences in retinal illuminance between the left and right eyes (cf. Table 2), The differences from the unfiltered eyes’ settings are greatest when two bright objects are seen against a dark background and the whole visual field is filtered for one eye (Column “a”). In this case, detectable changes of depth occur which, with the 95.7% filter, are equal to a disparity of some 100 arc seconds. But even when a 65% filter is used, visible changes of depth are detected that are the equivalent of a disparity of approximately 30 arc seconds.

If two white objects are viewed, Phenomenon 1 is reduced by half when, instead of the whole field of vision, only one eye’s view of a single object is filtered (Column “b”). Moreover, Phenomenon 2 is very much in evidence. If, however, one black and one white object are viewed, a distinct difference in the magnitude of Phenomenon 1 is not detectable when either the whole field of vision or only the object is filtered (Columns “d” and “e”).

Phenomenon 1 increases with the difference in luminance or, in other words, with the strength of one eye’s filter, and is detectible with all of the studied objects. The phenomenon itself, however, is substantially smaller when a black object is compared with a white one on a mid-luminous background (Columns “d” and “e”); it is even smaller, as already noted, when the signs are reversed and black stimuli are seen against a white background (Column “c”). Finally, the phenomenon is nearly invisible when gray stimuli are seen against a more luminous background (Column “f”) (cf. Table 5 under “c”).

#### Rule 4

As the width of the objects that have been subject to filtering increases, Phenomenon 1 at first increases but then remains constant (cf. Table 3). The relation between the two is not pronounced; it is most clearly recognizable when white objects are heavily filtered. With very narrow objects, Phenomenon 1 disappears almost completely, and it attains its highest value with objects having a horizontal retinal angle of one degree, which, even with wider objects, is not exceeded.

With respect to the dependence of Phenomenon 1 on the size of the object, it is necessary to make one observation about those objects that can be viewed without eye movements. On careful examination, these objects, viewed when one eye is filtered, appear to be turned on an axis that goes through the objects and is vertical to the direction of the observation in the medial plane. So what was said about Phenomenon 1 is valid, strictly speaking, only for the edges of the objects that are turned toward one another. This is the usual procedure. Rotation is no longer seen in Phenomenon 1 if the retinal angle of the object is so small that it is automatically localized in the mid-range depth of its vertical edges.

#### Rule 5

Phenomenon 1 increases with the contrast between the object and the background (cf. Tables 2 and 4). The dependency is only very stark when white objects are seen against a dark background, and it becomes more pronounced the more one eye is filtered.

#### Rule 6

Phenomenon 2 increases as the filtering is increased (cf. Table 5). At 65%, it is very small, that is, the equivalent of a disparity of about 10 arc seconds. With a filter of 95.7%, the visible change of depth corresponds to a disparity of some 50 arc seconds. As was already noted, when the corresponding image of the object is completely blotted out in one eye, the visible change of depth is substantially larger, but no longer easily measurable.

Given its nature, Phenomenon 2 can be noticed most clearly and with some certainty only if one of the stimuli—not the total field of vision—is filtered in one eye (Columns “a” and “b”). In Column “a,” Phenomenon 2 appears pure because Phenomenon 1 is not present due to the small size of the filtered object. But Phenomenon 2 does occur even when the whole field of vision is filtered if, as a result of the particular nature of the stimuli, the filtering primarily affects one of the stimuli, for example, the white one, when the other is black. This also applies to cases (cf. Table 2 Columns “d” and “e”) in which, even with respect to Phenomenon 1, there is hardly a difference whether one filters the whole field of vision or only the white object. Oddly enough, however, Phenomenon 2—or a phenomenon similar to it! —can also occasionally occur if both objects are equally luminous, for example, both gray (Column “c”). This may have to do with spatial differences across the retina, but that cannot be proved (cf. also Table 2 Column “f”).

The size of the object, luminance, and contrast seem to play no role in Phenomenon 2.

### Significance

Phenomenon 1 can easily be understood as a result of irradiation. It precipitates an increase in the size of the area of the brighter image and a corresponding decrease in darker areas, thus causing an apparent shift of the edge of the object in the direction of the darker image regions: Bright objects on a dark background appear larger than dark objects on a bright background. This apparent shift of the edge of the objects is, in general, the same in both eyes. But if one eye is filtered, then only the apparent edge of the image in this one eye will move toward the brighter parts of the image as a result of irradiation, contrast, and threshold value. This shift of the adjacent edges of two side-by-side objects toward one another in one eye is perceived, when observed binocularly, as a change in the relative depth of those edges.

Irradiation as an explanation of Phenomenon 1 (cf. Table 1) is supported by the fact that the effect of the filtering is equivalent to a mutual shifting of the edge of an object in the direction of the brighter regions (cf. Table 1). With a larger unstructured object, this spatial shift of the edges toward one another is transferred to the whole object and thereby determines its relative depth. When the edges that have been shifted are located far away from each other, then eye movements and the area of the image between the edges will certainly disturb their independent localization.

If the objects are smaller, and if both of the compared halves of the objects’ images fall into the area of the foveae, then the object in question will occasionally appear canted with respect to its depth (like a board viewed from its narrow side); but as the size of the object continues to decrease, one generally locates it in the mid-range of its lateral limit, that is, Phenomenon 1 disappears. Irradiation as an explanation of Phenomenon 1 can also account for the effects of filter absorbance and object contrast on the magnitude of the observed deviations. This is because the area of irradiation is 1—2 arc minutes and the largest disparities that correspond to the observed visible changes of depth with Phenomenon 1 are slightly less than 2 arc minutes.

If the explanation of Phenomenon 1 by irradiation is correct, it must have a correlate with monocular observation: If an eye is presented with a field of vision divided by a horizontal line separating it into two halves, in which two vertical edges that run perpendicular to this line are made coincident, then the setting that is produced when aligning the vertical edges should be contingent on the luminosity ratio between the two halves of the field of vision. This contingency was in fact observed in a coincidence-rangefinder. The deviations that occurred are of the same magnitude as Phenomenon 1.

There is, however, one apparent difficulty with the irradiation explanation, namely, that even when the contrast is held constant Phenomenon 1 is substantially smaller when black objects are seen against a white background than when white objects are seen against a black background. But this fact can be explained by adaptation. If one observes a field of vision with greater differences in luminosity, then, to a certain extent, adaptation is first and foremost determined by the brightest objects and then by the largest objects in the field of vision. In our case this means that when black objects are seen against a white background, the adaptation of the filtered eye is conditioned by the white background, because this is larger and brighter than the objects. The effect of the filter is thereby partially compensated for. On the other hand, when white objects are seen against a dark background, adaptation for the retinal area covered by the object’s image is, to be sure, determined by the white objects, but since they only cover a relatively small section of the field of vision, the images of the objects continually strike the parts of the retina that have adapted to the darker background as a result of eye movements. In this case, therefore, a compensation of the filtering by adaptation either does not occur at all or does so to a much lesser degree than in the first case.

It is certainly possible that further study of Phenomenon 1 will provide an answer to the still open question about bridging the gap between the histological understanding of the retina and the very low, physiologically determined threshold for the perception of a change in visual direction. Best (1900) believes it possible that a change in stimulation within strict limits induces the sensation of a difference in visual direction. But a change of stimulation that impacts the individual elements of the retina can be attained either by shifting the retinal image on individual cones or, as with the conditions of Phenomenon 1, by an attenuation of the light. The difference in stimulation, however, could also result from the fact that the directional value of the vertical rows of the retinal elements is determined by the number of the individual retinal elements that were stimulated within this. This view would follow Hecht’s (1930; Best, 1930) well-argued supposition that the number of stimulated retinal elements depends on retinal illuminance.

There is also another observation that might be easily explained in this context: In viewing two weakly illuminated objects that are located at equal distance from the observer, one notices great variation in their relative depth. This variation has nothing to do with the feeling of uncertainty that occasionally arises when conditions are unfavorable. It could be caused by the statistically detectable variations of the number of retinal elements (or of their light sensitivity) that are stimulated at the edges of the object’s image at a very low level of illumination.

In contrast to Phenomenon 1, Phenomenon 2 currently eludes an absolutely clear interpretation. Nevertheless, one may conclude from the transition of the phenomena, that is, from when the object of an image is partially filtered in one eye to when there is a complete disappearance of the object from that eye, that Phenomenon 2 is connected with the localization of monocularly viewed objects in a binocular field of vision. The literature seems not to have studied these phenomena yet. One can get a sense of their peculiarity if one places an object in the right or left part of an image of a stereoscope, which is therefore viewed monocularly, for example, a pencil. Such an object is localized with astounding certainty, usually behind the binocularly viewed partial images, if they are not subject to other aspects of depth perception, for example, occlusions, integration with other partial images, and so forth. But it is certainly not the case (as one might first suppose) that such monocularly viewed objects in a binocular field of vision are regularly localized at the visual egocenter.

This set of facts once again brings to mind the localization of double images to which Hering paid such great attention. By and large, double images are not localized at the visual egocenter when they are first observed but they are found at a point in space that lies near the real position of the object when seen as double images. After a certain period of time, however, the double images do move toward the visual egocenter. Each of the single images in a double image is that of a monocularly viewed object in a binocular field of vision; the essential difference vis-à-vis our experiments consists in the fact that the other single image is always still present in the other eye. The relationship of one to the other single images can obviously not be suppressed.

## Author Note

Edward T. Larkin and Wm Wren Stine are the translators for this paper.

## Notes

1 The Pulfrich effect was exactly predicted and explained by E. Mach without his having observed it (Dvořak, 1872).

2 The details of the apparatus used will be discussed elsewhere (*Zeitschrift für Instrumentenkunde*).

3 Translator’s Footnote: Münster uses the word “Winkelminute” (arc minute), but in the tables he refers to arc seconds.

## Appendix: Münster’s Post-War Years, Briefly, and Scientific Publications

With the end of World War II, Münster switched fields to begin a career in journalism (co-editor of *Frankfurter Hefte*) and broadcasting (managing editor of cultural and educational programming, Bavarian Radio, and then chief administrator of Bavarian Television). Hasselbring (1998) gives a brief but thorough biography of Münster in German.

A, probably incomplete, list of Münster’s scientific publications:

- Dierks, K., & Münster, Cl. (1933). Polarisationsoptische Studien am menschlichen Vaginalepithel [Polarization optical studies of the human vaginal epithelium]. *Archiv für Gynäkologie*, *152*, 1–12.
- Münster, Cl. (1932a). Unähnliche Abbildung und Ausmessung von Nichtselbstleuchtern. Ein Beitrag zum Äquivalenzprinzip [Divergent reproduction and measurement of non-self-illuminators. A contribution to the equivalence principle]. *Annalen der Physik, 407*, 619–644.
- Münster, Cl. (1932b). Über Anomalien bei Kathodenzerstäubung [On anomalies in cathode sputtering]. *Zeitsschrift für Physik, 75*, 716–722.
- Münster, Cl. (1933). Über die Ausmessung von Schwingungsellipsen bei elliptisch polarisiertem Lichte sehr großer Elliptizität [On the measurement of vibrating ellipses in elliptically polarized light with great ellipticity]. *Zeitschrift für Kristallographie – Crystalline Materials*, *86*, 325–334.
- Münster, Cl. (1934). Über die magnetische Doppelbrechung von Lösungen paramagnetischer Salze [On the magnetic birefringence of solutions of paramagnetic salts]. *European Physical Journal A*, *88*, 593–600.
- Münster, Cl. (1941). Ueber den Einfluß von Helligkeitsunterschieden in beiden Augen auf die stereoskopische Wahrnehmung [The effect of interocular brightness differences on stereoscopic perception]. *Zeitschrift für Sinnesphysiologie,* 69, 245–260.
- Szivessy, G., Dierkesmann, A., & Münster, Cl. (1933). Über die photographische Messung sehr schwach elliptisch polarisierten Lichtes im Ultravioletten [On the photographic measurement of very weakly, elliptically polarized light in the ultraviolet]. *Zeitschrift für Physik, 82*, 279–308.
- Szivessy, G., & Münster, Cl. (1928). Über eine Methode zur genauen Bestimmung der Auslöchungsstellungen [On a method to determine precisely the points of effacement]. *Zeitschrift für Physik, 47*, 357–361.
- Szivessy, G., & Münster, Cl. (1929). Über eine Methode zur Messung schwach elliptisch polarisierten Lichtes im Ultravioletten [On a method to measure very weakly, elliptically polarized light in the ultraviolet]. *Zeitschrift für Physik, 53*, 13–51.
- Szivessy, G., & Münster, Cl. (1931). Über eine Methode zur Messung schwach elliptisch polarisierten Lichtes im Ultravioletten [On a method to measure very weakly, elliptically polarized light in the ultraviolet]. *Zeitschrift für Physik, 70*, 750–757. [Supplementary material for Szivessy and Münster (1929).]
- Szivessy, G., & Münster, Cl. (1934). Über die Prüfung der Gitteroptik bei aktiven Kristallen [On testing optical gratings with active crystals]. *Annalen der Physik*, *412*, 703–736.
